# *Cynodon dactylon* and *Sida acuta* extracts impact on the function of the cardiovascular system in zebrafish embryos

**DOI:** 10.1016/S1674-8301(12)60017-7

**Published:** 2012-03

**Authors:** Rajaretinam Rajesh Kannan, Samuel Gnana Prakash Vincent

**Affiliations:** International Center for Nanobiotechnology (ICN), Center for Marine Science and Technology (CMST), Manonmaniam Sundaranar University, Rajakkamangalam, Kanyakumari Dist, Tamil Nadu 629502, India.

**Keywords:** cardiogenesis, small molecules, heart beat rate assay, blood flow velocity, zebrafish embryo

## Abstract

The aim of the present study was to screen cardioactive herbs from Western Ghats of India. The heart beat rate (HBR) and blood flow during systole and diastole were tested in zebrafish embryos. We found that *Cynodon dactylon (C. dactylon)* induced increases in the HBR in zebrafish embryos with a HBR of (3.968±0.344) beats/s, which was significantly higher than that caused by betamethosone [(3.770±0.344) beats/s]. The EC_50_ value of *C. dactylon* was 3.738 µg/mL. The methanolic extract of *Sida acuta (S. acuta)* led to decreases in the HBR in zebrafish embryos [(1.877±0.079) beats/s], which was greater than that caused by nebivolol (positive control). The EC_50_ value of *Sida acuta* was 1.195 µg/mL. The untreated embryos had a HBR of (2.685±0.160) beats/s at 3 d post fertilization (dpf). The velocities of blood flow during the cardiac cycle were (2,291.667±72.169) µm/s for the control, (4,250±125.000) µm/s for *C. dactylon* and (1,083.333±72.169) µm/s for *S. acuta*. The LC_50_ values were 32.6 µg/mL for *C. dactylon* and 20.9 µg/mL for *S. acuta*. In addition, the extracts exhibited no chemical genetic effects in the drug dosage range tested. In conclusion, we developed an assay that can measure changes in cardiac function in response to herbal small molecules and determine the cardiogenic effects by microvideography.

## INTRODUCTION

The zebrafish heart provides a useful vertebrate cardiovascular model with outstanding advantages, including genetic manipulability, optical accessibility and rapid development[1]. The zebrafish embryo is virtually transparent, which permits imaging of internal organs, especially the heart, using standard light microscopy. The embryonic zebrafish heart is the first organ to develop, which shows similarity to the embryonic human heart. Specific comparison has been drawn to show developmental parity between the 24 h post fertilization (hpf) zebrafish heart and the human heart at three weeks[2],[3]. In vertebrates, the heart precursors from both sides of the embryo move medially during gastrulation and reside on either side of the midline as part of the lateral mesoderm. The bilateral heart primordia then fuse at the midline and form the primitive heart tube, consisting of two concentric tubes. The outer layer, the myocardium, will form the heart muscles and the inner layer, the endocardium, will become the inner lining of the heart[4]-[6]. Zebrafish provides an ideal model for the study of vertebrate organogenesis[7]. The model system is finding utility in the investigation of compounds that have potential relevance to both human and environmental health, as well as biomedicine[8]. The heart is an ideal model system for both embryological and genetic approaches to organogenesis since it develops early, and it is readily accessible for observation and manipulation.

The heart is composed of a limited number of cell types, and has been well described with regard to particular developmental milestones[4]. The larval zebrafish at 72 hpf is approximately 1 mm in length, and as such many embryos can live for several days in a single well of a 96- or even 384-well plate. Image processing techniques, such as digital subtraction analysis, have been used to measure circulatory flow and cardiac function and defined normal values through development[9]-[11]. Drug effects on cardiac functions, including heart rate, rhythmicity, contractility and circulation are visually assessed in zebrafish at 48 hpf using a dissecting microscope. At 22 hpf, the cardiovascular system is fully functional and exhibits a complex repertoire of ion channels and metabolic processes. Zebrafish *ERG* (ether-a-go-go related gene) is expressed in the early stages of zebrafish development and the amino acid sequence of the pore-forming domain of zebrafish ERG and human ERG are 99% conserved[12].

The zebrafish has been shown to be an excellent model for assessing drug-induced cardiotoxicity[13],[14]. Although zebrafish and mammalian hearts differ in structure and zebrafish lacks a pulmonary system, they exhibit similar functional characteristics, including: 1) blood flows from a major vein atrium into an atrium; 2) blood moves through a muscular ventricle for delivery to the aorta; 3) valves direct blood flow; 4) a specialized endocardium musculature drives a high-pressure system; 5) an electrical system regulates rhythm; 6) heart beat is associated with pacemaker activity[12],[15]. The cardiovascular system can be easily visualized in the embryo and the effect of herbal small molecules on cardiac cycle can be investigated. The present study was designed to screen select herbal extracts that affect the cardiac cycle and the blood flow rate in zebrafish.

## MATERIALS AND METHODS

### Herbal sample collection and preparation

Thirty medicinal herbs (120 extracts) (***Supplementary table***) were collected from Shenbagaramanputhoor, Western Ghats of Kanyakumari, India. All the herbs were washed in tap water and rinsed with distilled water to remove the minerals on the plant materials and shade dried for 25-30 d. The list of the medicinal herbs is shown in the ***Supplementary table***. The dried herbs were powdered into 1 mm particle size in a mixer grinder. Ten g of the powdered samples were filtered and extracted in a Soxhlet apparatus using hexane, chloroform, acetone and methanol, based on their increasing polarity. After extraction, the four different solvent extracts were allowed to evaporate and then concentrated in a vacuum concentrator (Eppendorf, Hamburg, Germany). The concentrated extracts were transferred to the Eppendorf tubes (2 mL) and stored at 4°C[16].

### Breeding and maintenance of zebrafish

Zebrafish were bred and maintained in the Fish Culture Facility of the International Center for Nanobiotechnology, Center for Marine Science and Technology, Manonmaniam Sundaranar University, India. Zebrafish were maintained in 30 L tanks at 28°C with 14 h/10 h light/dark cycle. Following successful breeding, eggs were sieved through the mesh, and subsequently collected from the bottom of the tanks. Zebrafish were maintained as described previously[17]. Zebrafish embryos were raised in E3 medium (5 mmol/L NaCl, 0.17 mmol/L KCl, 0.4 mmol/L CaCl_2_ and 0.16 mmol/L MgSO_4_ in 1 L ddH_2_O). Eggs containing dead or obviously weak embryos were removed. The remaining embryos were used, usually within 2 hpf, for the developmental toxicity assays. Embryos were raised in 10 mmol/L HEPES-buffered E3 medium in a dark incubator at 28°C until 60 hpf. Eggs containing dead or obviously weak embryos were removed[18],[19]. The study protocol was approved by the local institutional review board at the authors' affiliated institution and animal study was carried out in strict accordance with the established institutional guidelines.

### Pharmacological manipulations

One mg/mL of the stock solution was prepared. For assessment of the effects of herbal small molecules on the physiology of developing zebrafish embryos, the phytomolecules were dissolved in ERS to final concentrations of 1-100 µg/mL from concentrated stock solutions. Embryos were pipetted into the solution in a minimal volume and then imaged. Parallel control was made to compare the physiological effects of the extract. Zebrafish embryos at 72 hpf were used for incubation and imaged subsequently as described by Shin *et al*.[20].

### Image acquisition and quantitative image analysis

Zebrafish embryos were anesthetized in 1% MS-222 (Sigma, St. Louis, MO, USA) and individual embryos were checked for lateral positioning and allowed to acclimate to the microscopic illumination for 1 min. At this developmental stage, the embryos are still transparent allowing excellent visibility of internal structures including the heart and circulation. Video microscopy was performed on a light microscope (Motic) with objective lens of either 4× or 10×. Sequential images of the heart and dorsal aorta were obtained with the embryo positioned on its side from the lateral position at 25 frames per second (fps) at a shutter speed of 0.04 frames/s (4 cs). To ensure a comparable plane of focus for ventricular analysis, zebrafish hearts were imaged in a standardized lateral position, with the ventricle clearly visible in the plane of focus throughout the cardiac cycle. The atrium usually lay outside the plane of focus. For imaging of tail circulation, the cloaca was chosen as a uniform anatomic landmark for dorsal aortic images. Acquired movies were stored as serial still images on fixed optical media for subsequent off-line analysis. To quantitate the physiological parameters of cardiovascular performance in the zebrafish, Adobe Premiere 6.5 and ImageJ (NIH) were used for analyses. Sequential still frames were analyzed to identify the frames as systolic or diastolic. The endocardial boundary was traced, and the area of the region defined by this trace was measured. Three sequential cardiac cycles (pairs of systolic and diastolic frames) were recorded. The heart beat rate (HBR)/cardiac cycle in embryonic zebrafish was calculated.

### Quantitative measurement of blood flow velocity

The blood flow in the dorsal aorta was calculated by sequential still images by highlighting individual erythrocytes as they moved between frames. The entire cardiac cycle was analyzed by the movement of blood flow in the aorta. One somite region of the embryo was measured as 30 µm per somite, the movement was calculated based on the movement of blood cells between the somites and the distance was calculated in a series of 10 frames of images edited in the Adobe Premiere 6.5.

### Determination of the LC_50_ and EC_50_ of the herbal extracts

The toxicity to the zebrafish embryo was tested according to OECD[21]. Fertilized eggs were selected for the test and placed in 24-well plates. In each well, ten eggs were exposed to 1 mL of the test solutions containing 1% dimethyl sulfoxide (DMSO) in aerated water. Each substance was tested in 10-100 µg/mL of the methanolic extracts from *Cynodon dactylon* (*C. dactylon*) and *Sida acuta* (*S. acuta*). All experiments were repeated three times. Eggs were incubated at 26°C. After 24 hpf, eggs were examined using a light microscope. The parallel control was exposed to the 1% DMSO solvent only. After 96 h of treatment, the LC_50_ values were determined using probit analysis performed in the statistical software SPSS 16.0, and the EC_50_ values were calculated using the 4-parameter logistic nonlinear regression model performed in the software SigmaPlot 10 (Systat software Inc.).

### Statistical analysis

Each experiment was performed at least three times, and all values were described as mean±standard deviation of triplicate assays. Differences of the results were tested for the statistical significance using Student's *t*-test. *P* values < 0.05 were considered statistically significant.

## RESULTS

### Analysis of cardiac cycle

**Fig. 1:**
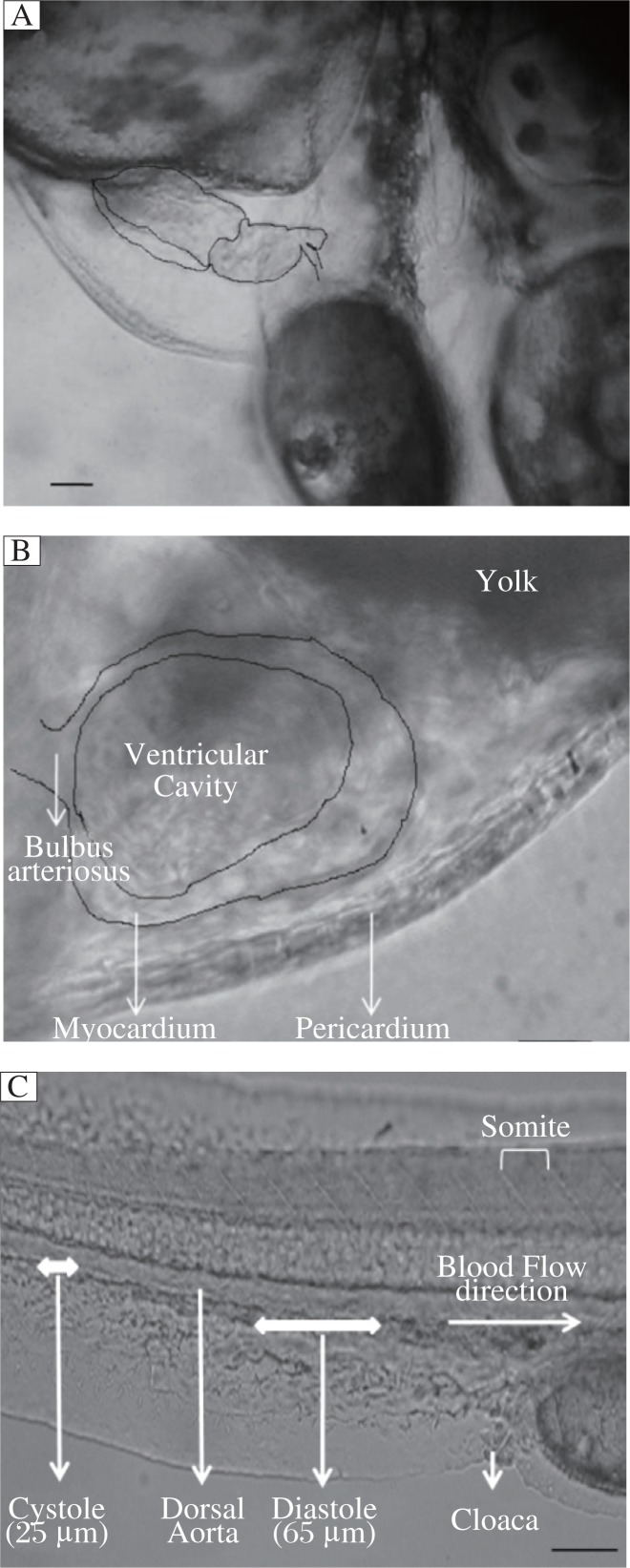
Anatomy of the zebrafish heart. The ventricle empties rostrally with each contraction through the bulbus arteriosus at the ventral side of the zebrafish. The pericardium lies adjacent to the myocardium on the outer curvature of the ventricle. The ventricular cavity was identified by the blood in it. A: The lateral view of the normal heart of a zebrafish embryo at 3 d post fertilization. Scale bar = 10 µm. B: The ventricular changes in a cardiac cycle. Scale bar = 10 µm. C: Schematic presentation of measurement of blood flow velocity in zebrafish embryos. Scale bar = 30 µm.

**Fig. 2:**
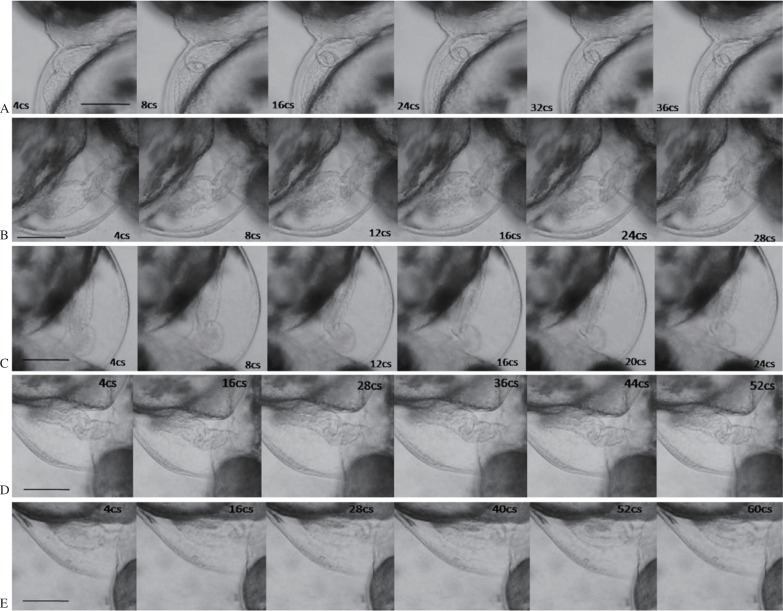
Sequential frames of cardiac cycles in zebrafish (one systole and diastole) in Adobe Premiere 6.5. A: Sequential frames of the duration of one cardiac cycle in zebrafish calculated in untreated embryos. Scale bar = 30 µm. B: Sequential frames and the duration of one cardiac cycle in zebrafish treated with betamethosone (10 ng/mL). Scale bar = 30 µm. C: Sequential frames and duration of one cardiac cycle in *C. dactylon* extract at 6 µg/mL. Scale bar = 30 µm. D: Sequential frames and duration of one cardiac cycle in 3 d post fertilization zebrafish embryos treated with nebivolol at 23.3 ng/mL. Scale bar = 30 µm. E: Sequential frames and the duration of one cardiac cycle in 3 d post fertilization zebrafish embryos treated with 3 µg/mL *S. acuta* extracts. Scale bar = 30 µm. cs: centisecond.

**Fig. 3:**
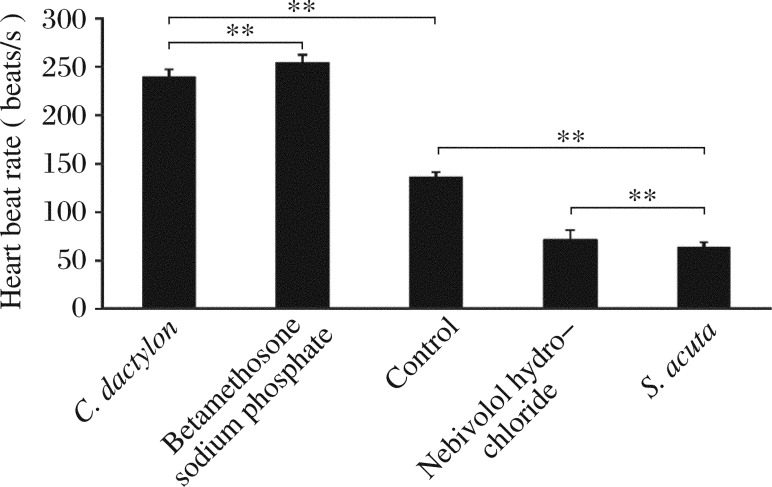
Heart beat rates of zebrafish embryos at 3 d post fertilization treated with *C. dactylon, S. acuta* extracts and commercial molecules and of the untreated zebrafish embryos. Each value was expressed as mean±SD of three replicates, **P < 0.01 (revealed by *t*-test and subsequent posthoc multiple comparisons with SNK test).

The zebrafish heart anatomy and blood flow are shown in ***Fig. 1***. The methanolic extract of *C. dactylon* decreased the duration of systole and diastole, resulting in an increase in HBR. One second of video consisted of 25 frames of images. The HBR was calculated based on the duration of one cardiac cycle and it was (2.685±0.160) beats/s in the control and (3.968±0.344) beats/s for methanolic extract of *C. dactylon* (6 µg/mL), and there was a significant difference (*P* = 0.002,1), while the latter was greater than (3.770±0.344) beats/s of the commercial drug betamethosone sodium phosphate at a concentration of 10 ng/mL (*P* = 0.003,9). The methanolic extracts of *C. dactylon* induced reduction in the duration of cardiac cycle (***Fig. 2B*** and ***2C***). The HBR for 1 min was calculated at increasing concentrations (2-10 µg/mL) of the methanolic extracts of *C. dactylon*. The quantification of HBR for *C. dactylon*, betamethosone sodium phosphate and the untreated is shown in ***Fig. 3***. The EC_50_ value was 3.738 µg/mL (*P* = 0.000,1) for *C. dactylon* calculated using SigmaPlot 10. Treatment of *S. acuta* extracts at 3 µg/mL led to decrease in HBR at (1.877±0.079) beats/s (*P* = 0.000,7), which was approximately equal to (1.667±0) beats/s by the positive control nebivolol hydrochloride at 23.3 ng/mL (*P* = 0.000,2), and increase in the duration for one cardiac cycle in the embryos (***Fig. 2D*** and ***2E***). Meanwhile, a HBR of (2.685±0.160) beats/s in the 3 dpf embryos was observed (***Fig. 2A***). The EC_50_ value was 1.195 µg/mL calculated using SigmaPlot 10 (*P* = 0.000,1). The quantification of decreased HBR in *S. acuta*, nebivolol hydrochloride and the untreated embryos is shown in ***Fig. 3***. The duration of cardiac cycle and the anatomical changes in the heart of 3 days post fertilization (dpf) zebrafish embryos are presented in ***Fig. 2***.

### Blood flow velocity

**Fig. 4:**
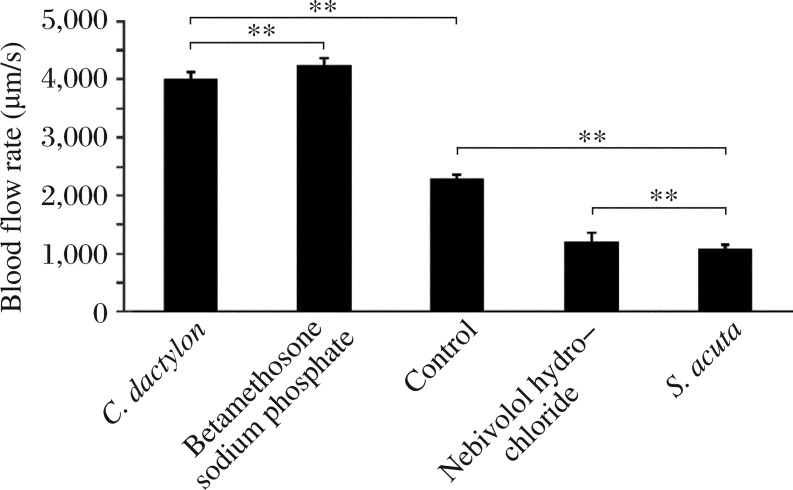
Blood flow velocity of 3 d post fertilization zebrafish embryos for one cardiac cycle treated with *C. dactylon, S. acuta*, the positive controls and of the untreated zebrafish embryos at 3 d post fertilization. Each value was expressed as mean±SD of three replicates, **P < 0.01 (revealed by *t*-test and subsequent post-hoc multiple comparisons with SNK test).

Blood flow was calculated based on the movement of cells in a series of images obtained from the digital video editing software Adobe Premiere 6.5. The images were calculated based on the movement of blood cells among somites in each frame and the length of the somite was found to be 30 µm. The representative blood flow is presented in ***Fig. 1C***. The movement of single blood cell was calculated. The systolic blood flow velocity was (583.333±72.169) µm/s for the control and (1,416.667±72.169) µm/s for *C. dactylon* extracts at a concentration of 6 µg/mL (*P* = 0.000,07), whereas (1,333.333±72.169) µm/s for the positive control betamethosone sodium phosphate (*P* = 0.000,1). *C. dactylon* extracts caused a 142.2% increase in blood flow during systole, which was higher than that caused by betamethosone (128.6%). The blood flow was (250±0) µm/s for *S. acuta* extracts at a concentration of 3 µg/mL (*P* = 0.000,7), which was significantly less than (291.667±72.169) µm/s by the positive control nebivolol (*P* = 0.003,8). *S. acuta* extracts induced a 57.14% decrease in blood flow during systole, which was greater than that by nebivolol (50%). The diastolic flow velocity was (1,708.333±72.169) µm/s for the control and (2,833.333±72.169) µm/s for *C. dactylon* extracts (*P* = 0.000,02) at 6 µg/mL, while betamethosone yielded a flow velocity of (2,666.667±72.169) µm/s (*P* = 0.000,04). *C. dactylon* extracts caused a 65.9% increase in blood flow during diastole, which was higher than that by the positive control (56.1%). The blood flow velocity was (833.333±72.169) µm/s for *S. acuta* extracts at the concentration of 3 µg/mL (*P* = 0.000,06), which was similar to that (916.667±72.169) µm/s by nebivolol (*P* = 0.000,08). A 51.2% decrease during diastole was observed following treatment with *S. acuta* extracts, which was higher than that post-treatment with the commercial molecule (46.3%). The blood flow velocity for a cardiac cycle (systole and diastole) was (2,291.667±72.169) µm/s for the control, (4,250±125) µm/s for *C. dactylon* extracts (*P* = 0.000,009), and (4,000±125) µm/s for betamethosone sodium phosphate (*P* = 0.000,016). For *S. acuta*, the blood flow velocity was (1,083.333±72.1688) µm/s (*P* = 0.000,016), which was significantly less than that (1,208.333±144.337,6 µm/s) of nebivolol (*P* = 0.000,156). Blood flow velocities during systole and diastole are shown in ***Fig. 4*** and ***Table 1***, and there were significant differences in blood flow detected by the Student's *t*-test. *C. dactylon* extracts caused an 85.5% increase in blood flow in a cardiac cycle, which was higher than that caused by betamethosone (74.5%); while a 52.7% decrease in blood flow was caused by *S. acuta* extracts, which was lower than that by nebivolol (57.3%).

**Table 1:** Blood flow velocity in a cardiac cycle of zebrafish at 3 d post fertilization treated with *C. dactylon* and *S. acuta* extracts

Drags	Cardiac cycle	
	Systole	Diastole
Control	583.333 ± 72.168	1,708.333 ± 72.168
Betamethosone (10 ng/mL)	1,333.333 ± 72.168	2,666.667 ± 72.168
*C. dactylon* (6 µg/mL)	1,416.667 ± 72.168	2,833.333 ± 72.168
Nebivolol (23.3 ng/mL)	291.667 ± 72.168	916.667 ± 72.168
*S. acuta* (3 µg/mL)	250 ± 0	833.333 ± 72.168

(µm/s)

### Organogenesis of the heart

**Fig. 5:**
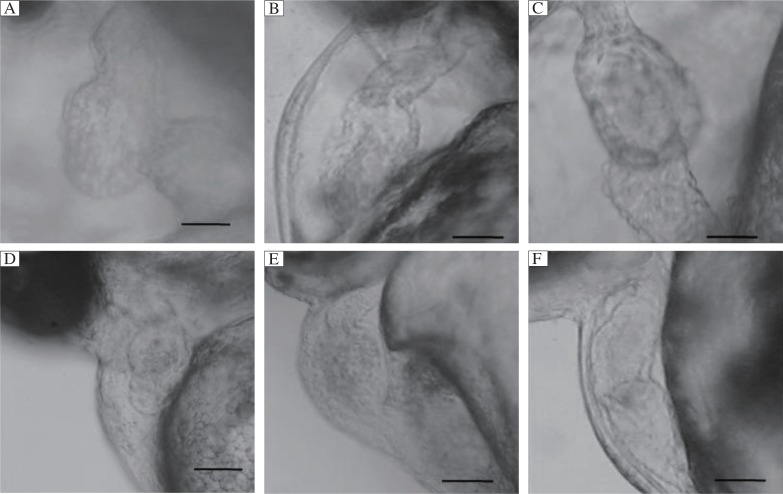
Cardiogenic effect of the methanolic extracts of *C. dactylon* (30-40 µg/mL) and *S. acuta* (20-30 µg/mL) at 3 days post fertilization zebrafish embryos. A: S-like structural deformity was observed in the ventricle following treatment with *C. dactylon*. Scale bars = 10 µm. B: Untreated embryos (parallel control). Scale bars = 5 µm. C: Presence of bradycardia (tubular heart) and ventricular bulging were observed (*C. dactylon* extract). Scale bars = 10 µm. D: Cardiotoxic effects of the methanolic ex-tracts of *S. acuta* showing abnormal heart when compared to the control. Scale bars = 5 µm. E: Cardiac edema in both the atrium and ventricle with necrosis was observed in the yolk sac region. Scale bar = 5 µm. F: Untreated embryo (parallel control). Scale bars = 5 µm.

Cardiogenic effects were observed in higher concentrations of the two herbs. An abnormal and S-like structure was observed in the ventricle of the embryonic heart, which clearly demonstrated the cardiogenic effects of *C. dactylon* extracts (30-40 µg/mL) at its LC_50_/higher concentrations in the embryonic heart of zebrafish at 3 dpf (***Fig. 5A***) when compared to the untreated embryos (***Fig. 5B***). Additionally, a tubular heart with mild bulging of the ventricle was observed in *C. dactylon*-treated 3 dpf embryos (***Fig. 5C***). Abnormal heart was found in 3 dpf embryos treated with 20-30 µg/mL *S. cuta* extracts compared to the untreated embryos (***Fig. 5E*** and ***5F***). Cardiac edema was observed in both the atrium and ventricle of the embryonic heart, and necrosis was detected in the yolk sac region (***Fig. 5E***). Cardiac arrhythmia (inconsistent blood flow) with reduced HBR was observed while they were treated with the two herbs at their LC_50_ and higher concentrations. Notably, both herbal extracts induced no *has*- and *small*-mutant phenotypes in the cardiac region of the embryos.

### Determination of LC_50_ and EC_50_ values

The LC_50_ was 32.608 µg/mL (95% confidence interval: 25.76-38.677 µg/mL) for *C. dactylon* determined using probit analysis, and 20.877 µg/mL (95% confidence interval: 14.411-26.573 µg/mL) for *S. acuta*. The EC_50_ value of inducing cardiac cycle was 3.7381 µg/mL (*P* = 0.000,1) for *C. dactylon*, while the EC_50_ value of decreasing cardiac cycle was 1.195 µg/mL (*P* = 0.000,1) for *S. acuta*.

## DISCUSSION

The purpose of this study was to develop an assay to measure changes in cardiac function in response to herbal small molecules and to determine the cardiogenic effects using microvideography. Zebrafish is a good model organism to study the cardiovascular system and organogenesis. The organogenesis in the embryonic development of zebrafish has been investigated in previous studies[22],[23]. Small molecule perturbants of physiological function have been isolated using assays that take particular advantage of whole animal-based *in vivo* screens. For example, an assay has been developed that allows the heart rates of zebrafish larvae in 96- or 384-well plates to be determined automatically using a robotic microscope coupled with a digital video camera and image processing software[24]. Such an assay can easily be used to identify novel compounds affecting cardiac physiology. Small fish embryos can be assayed in microplates to identify bioactive molecules that perturb phenotypic traits in the heart[25]. Based on the previous studies and significance of small molecule screening, the effects of the herbal extracts of *C. dactylon* and *S. acuta* on the cardiac cycle and blood flow velocity were explored in the embryos of zebrafish at 3 dpf. The methanolic extract of *C. dactylon* induced changes in the cardiac cycle (systole and diastole) and blood flow in the embryonic zebrafish at 3 dpf (74–80 hpf). Similarly, atropine induces an increase in HBR caused by the stimulation of cardiac cycle[26]. Small molecules from *S. acuta* decreased heart rate and similar effects were observed in propranolol, which also decreased heart rate in zebrafish embryo.

The cardiac regulation of zebrafish in response to sudden onset stimuli is functionally similar to that of mammals, suggesting that these responses and their underlying mechanisms are functionally conserved[26]. The pharmacological effects on cardiac systolic and diastolic function have not been widely studied[7]. Flow of blood cells is clearly seen inside blood vessels in zebrafish embryos once circulation begins at 24 hpf. The flow is pulsatile, with a rhythm of fast and slow movement[27]. Recently, Shin *et al*.[20] developed a suite of software tools to assess cardiovascular physiology in living zebrafish embryos. In this study, blood flow velocity changes in embryos treated with herbal extracts and commercial drugs (positive control) and the untreated embryos were detected. The microvideography was used to visualize all the treated embryos during systole and diastole of each cardiac cycle. The results showed that the herbal small molecules affected the cardiovascular system by stimulating the cardiac cycle and reducing blood flow velocity. Many studies have demonstrated the conservation of small-molecule response within the cardiovascular system of the developing zebrafish embryo and suggested that several prototypic drug-responsive pathways are intact in zebrafish embryos even at an early developmental stage. The important role of the Na^+^-K^+^-ATPase α1β1-subunit in cardiac development has been demonstrated through mutations that cause the *heart* and *mind* and *small heart* defects[28],[29]. We detected the cardiac mutation stimulated by the methanolic extracts of *C. dactylon* and *S. acuta*, but did not find the effects of *small* and *has* mutations. Some other mutations of tubular heart and changes in cardiomorphology have been observed for both extracts after 12 h. Loss of α1β1-subunit is associated with small heart, diminished cardiac contraction, and absence of circulation[28], which affects the physiology and development of the heart but does not affect HBR. In the current study, no *has* and *small* mutations were detected in the small molecules from *C. dactylon* and *S. acuta*[18]. Bradycardia was observed following treatment with the *C. dactylon* extract and the LC_50_ value was determined. The antagonist stropin has also been demonstrated to induce bradycardia[26]. Drug-induced cardiotoxicity remains a great challenge to patients, physicians, researchers and regulatory agencies around the world[30]. Many commercial drugs exhibit cardiotoxic effects and affect the cardiac cycle in zebrafish models[24]. In the past few years, zebrafish has emerged as an important model organism for small molecule discovery[31]. The powerful genetics of zebrafish and optical methods will provide new opportunities in broad areas of physiological, developmental and pharmacological cardiovascular research. In this study, these two herbal molecules showed no toxic effects in the drug dose. However, the two herbs induced significant increase and decrease in cardiac cycle compared with the commercial drug. Hence, the phytomolecules from the herbal extracts can be structurally characterized to investigate the efficacy of the molecules in future animal experiments.
